# Development and implementation of a lifestyle intervention to promote physical activity and healthy diet in the Dutch general practice setting: the BeweegKuur programme

**DOI:** 10.1186/1479-5868-7-49

**Published:** 2010-05-26

**Authors:** Judith HM Helmink, Jessie JM Meis, Inge de Weerdt, Femke N Visser, Nanne K de Vries, Stef PJ Kremers

**Affiliations:** 1 School for Nutrition, Toxicology and Metabolism (NUTRIM), Department of Health Promotion, Maastricht University, the Netherlands; 2 Netherlands Diabetes Federation, Amersfoort, the Netherlands; 3 Netherlands Institute for Sport and Physical Activity, Bennekom, the Netherlands; 4 School for Public Health and Primary Care (CAPHRI) and School for Nutrition, Toxicology and Metabolism (NUTRIM), Department of Health Promotion, Maastricht University, the Netherlands

## Abstract

**Background:**

The number of patients with diabetes is increasing. BeweegKuur (Dutch for 'Exercise Therapy') is a Dutch lifestyle intervention which aims to effectively and feasibly promote physical activity and better dietary behaviour in primary health care to prevent diabetes.

**Methods:**

The goal of this paper is to present the development process and the contents of the intervention, using a model of systematic health promotion planning. The intervention consists of a 1-year programme for diabetic and prediabetic patients. Patients are referred by their general practitioner (GP) to a lifestyle advisor (LSA), usually the practice nurse or a physiotherapist. Based on specific inclusion criteria and in close collaboration with the patient, an individual exercise programme is designed and supervised by the LSA. This programme can be attended at existing local exercise facilities or (temporarily) under the supervision of a specialized exercise coach or physiotherapist. All participants are also referred to a dietician and receive diet-related group education. In the first pilot year (2008), the BeweegKuur programme was implemented in 7 regions in the Netherlands (19 GP practices and health centres), while 14 regions (41 GP practices and health centres) participated during the second year. The aim is to implement BeweegKuur in all regions of the Netherlands by 2012.

**Discussion:**

The BeweegKuur programme was systematically developed in an evidence- and practice-based process. Formative monitoring studies and (controlled) effectiveness studies are needed to examine the diffusion process and the effectiveness and cost-effectiveness of the intervention.

## Background

Commissioned by the Dutch Ministry of Health, Welfare and Sports (VWS), a lifestyle intervention called 'BeweegKuur' was developed by the Netherlands Institute for Sport and Physical Activity (NISB)[1]. The BeweegKuur is a lifestyle intervention tailored to the individual needs of patients, focusing on a change in physical activity behaviour and dietary behaviour, to support the prevention and treatment of type 2 diabetes mellitus. The intervention is developed with the aim to become an effective and feasible primary health care based intervention, which in time can be reimbursed under the Dutch basic health insurance scheme. The current paper describes the rationale for the development of the BeweegKuur programme, as well as its development and contents. It also outlines the design of a formative evaluation study of the pilot implementation.

**Figure 1:**
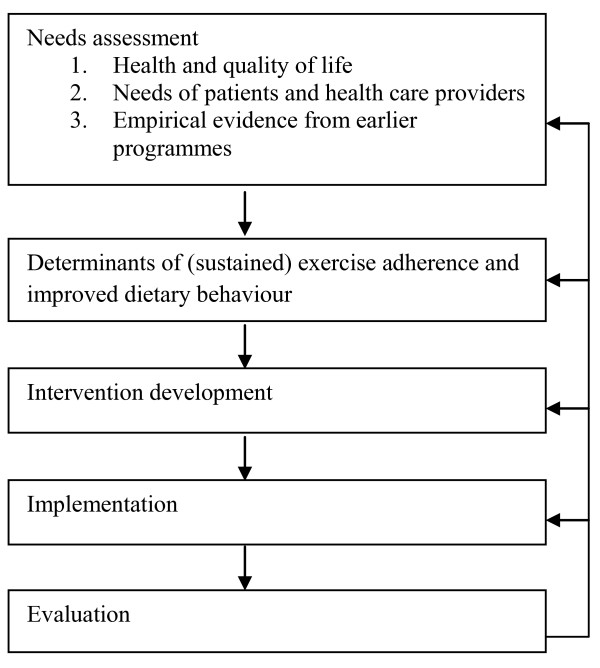
**Model of systematic intervention planning and development**.

Effective interventions should be based on the model of systematic intervention planning and development (Figure 1, see also [2,3]). The first step of this planning model comprises a needs assessment. This step involves gathering both quantitative (e.g., literature review) and qualitative (e.g., in-depth interviews) information regarding the needs for intervention development. The needs assessment starts by reviewing health and quality of life. It also reviews personal (i.e. patient-related) and environmental (e.g., health care professionals) factors involved in unhealthy lifestyle, as well as empirical evidence on existing primary health care interventions. The second step of the model explores the determinants of (sustained) exercise adherence and improved dietary behaviour. This is followed by the development of a prototype for the intervention (step 3) and its pilot implementation (step 4). All these steps should be evaluated in the fifth step. With respect to the prototype development and pilot implementation, the evaluation is typically and preferably of a formative nature [4]. The structure of this paper follows the steps of this planning model.

### Step 1: Needs Assessment

#### Health and quality of life

Approximately 740,000 people in the Netherlands were diagnosed with diabetes in 2007, 90% of whom had type 2 diabetes [5], while 250,000 to 740,000 people are estimated to be unaware that they have the disease [5]. Additionally, approximately 900,000 people aged 60 years and older suffer from early-stage diabetes (Impaired Glucose Tolerance: pre-diabetes) [6]. The number of Dutch citizens with diabetes is expected to have doubled by 2025, partly due to the ageing population and the increasing number of overweight people [6]. At least 40% of people with type 2 diabetes suffer from chronic complications, such as cardiovascular diseases, neuropathy, retinopathy and renal failure [7]. These types of complications particularly limit their mobility and, therefore, their ability to be physically and socially active, resulting in a reduced ability to live independently and in a lower quality of life [8]. Physical inactivity, unhealthy dietary behaviour and obesity play a significant role in the development of type 2 diabetes [9]. Currently, at least five million people in the Netherlands are overweight and physically inactive [6]. It has been argued that the greatest benefit to health can be achieved by getting physically inactive people with diabetes to become active, which can delay the development of complications in the long term and support and postpone pharmaceutical treatment [10,11]. The advantageous effects of exercise include changes in body composition and a decrease in blood pressure. Exercise also results in favourable effects on the regulation of the blood glucose level [12]. Finally, exercise as well as healthy dietary behaviour are not only important in the treatment of type 2 diabetes, but also decrease the risk of developing diabetes, and are therefore an important primary prevention measure [13].

#### Needs of patients and primary health care providers

**Figure 2:**
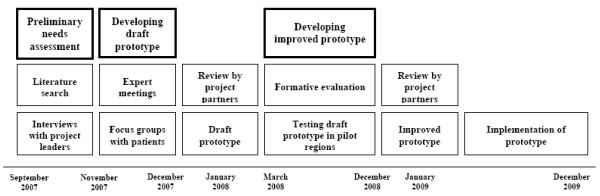
**Timeline of the development and implementation of the BeweegKuur programme**.

Before developing a prototype of the BeweegKuur intervention, a preliminary needs assessment was carried out (see Figure 2). A literature search was used to explore existing lifestyle interventions, national as well as international [14]. Based on this literature review, in-depth interviews were conducted with leaders of Dutch national projects relevant to the BeweegKuur programme. The outcomes of these interviews were used to develop the first outline of the intervention. During its development, the outline was discussed with primary health care providers as well as patients by means of face-to-face interviews and focus group sessions. The goal of this discussion process was to see how the draft prototype should be adjusted to fit the needs of both patients and health care professionals. The first step in the process consisted of interviews with project partners and specialists to assess whether the prototype was feasible and to discuss improvements to the prototype. In addition, four expert panels were formed in different regions in the Netherlands, each including eight to fourteen people, from different disciplinary backgrounds and with or without experience with lifestyle interventions. The outcomes of these expert panel meetings were incorporated in an improved version of the prototype.

Three focus groups with diabetic and prediabetic patients were then organized to assess the views of diabetic or prediabetic patients regarding the care they would expect, the role of health professionals and the perceived need for the BeweegKuur programme. The main outcomes of the focus group sessions were that referral to BeweegKuur by a general practitioner (GP) would provide additional motivation for patients, which is consistent with previous research [15]. The patients emphasized the importance of close collaboration between the different health care professionals and their need to feel safe with these professionals. Other issues included the importance of a smooth transition to existing local exercise facilities and of low costs. Finally, the patients indicated that they would be stimulated by noticeable results, for example initial loss of weight or an improvement in blood glucose levels.

The results of the focus groups were confirmed in three interviews with patients who were participating in an intervention similar to BeweegKuur. Important aspects they mentioned were close collaboration between health care professionals, personal guidance by a health care professional, a clear structure and exercising in a group with peers. The main reason for attending the lifestyle intervention appeared to be achieving weight loss. The opinions of health care professionals regarding lifestyle interventions were explored in nine face-to-face interviews with GPs, physiotherapists and lifestyle advisors (LSA) who were already participating in a lifestyle intervention. The health care providers affirmed the importance of lifestyle interventions. Important topics they mentioned included the crucial role of motivating patients to start and continue the intervention, and working in a multidisciplinary team.

#### Empirical evidence from existing lifestyle interventions

Various relatively controlled lifestyle interventions to prevent type 2 diabetes (i.e. highly supervised and executed within a research context) have been developed and evaluated in the Dutch context (e.g., Study on Lifestyle Intervention and Impaired glucose tolerance Maastricht (SLIM) [12,16], as well as internationally (e.g., the Diabetes Prevention Program (DPP) [17] and the Finnish Diabetes Prevention Study (DPS) [10]). Although details differ between the various studies in terms of outcomes and purposes, the general purport is that a controlled lifestyle intervention to improve physical activity and dietary behaviour has a positive effect on the health of participants [18]. However, it is not self-evident that relatively controlled lifestyle interventions will also be suitable for wider implementation in practice. The following paragraphs review lifestyle interventions delivered by primary health care centres all over the world and specifically in the Netherlands.

To increase patients' physical activity, interventions have been set up in primary health care settings in various countries, such as the UK [19], Finland [20,21], the United States [22], New Zealand [23] and Australia [24]. The typical conclusion from these types of intervention studies is that their effectiveness is limited. A meta-analysis showed that 17 inactive adults would have to be referred to an intervention to result in one of them starting to exercise for 30 minutes a day, with at least moderate intensity, on a minimum of five days a week [19]. A potential cause of the limited effectiveness relates to high attrition rates, because the intervention interferes with patients' other activities, is not in line with their capabilities [25] or is not designed and implemented so as to result in *sustained *behaviour change.

Previous studies have revealed various important implementation issues. An active patient role is required, and GPs should take enough time to convince patients that the intervention is beneficial and safe. 'Exercise-on-prescription' interventions were generally perceived as feasible by participating GPs and patients who voluntarily took part in the intervention. However, little is known about the degree of acceptance and feasibility for patients and GPs who refused to participate in an intervention, and for GPs who were not motivated to implement the intervention. GPs also stated that it is important for them to receive help from the intervention designers in terms of the implementation [26]. This is in line with the results of the implementation of the PACE intervention (Physician-based Assessment and Counselling for Exercise) in the US. More than half of the GPs indicated that, without the training, they would not have been able to introduce the intervention properly [22]. In Australia, research has shown that a prescription for exercise by a GP accompanied by written material led to an increase in self-reported physical activity by inactive individuals within a short period of time. However, this research also showed that a prescription for exercise alone did not result in more physical activity [24]. For example, GPs indicated that they preferred presenting patients with written exercise objectives rather than verbal ones, which also made the GPs feel comfortable about discussing physical activity with patients and prescribing exercise [24]. Although the GPs who participated in the study supported the concept, they indicated that the lack of time presented an immediate and significant obstacle, which might be overcome by suitable training and materials [23].

Factors that could discourage participants from exercising include high costs and the distance to exercise facilities; embarrassment about physical appearance; religion and culture; lack of time; inability or unwillingness to arrange a babysitter or attend during evening hours; and lack of support from the social environment [27]. Another barrier to participation in an exercise-on-prescription intervention could be low self-efficacy. External obstacles were also identified, such as an intimidating exercise environment, inadequate supervision during exercise and unfavourable opening hours [19]. Referral by a GP was an important motivation to participate because it forms a strong incentive and provides a legitimate reason for starting to exercise [27]. In the Netherlands, various initiatives have been set up in primary health care settings to increase patients' physical activity behaviour, with names like Big!Move [28,29], Exercise on Prescription [27,30] and 'From Complaint to Strength' [31]. The results of these projects were generally favourable in terms of self-reported effect indicators. Participants indicated that they had increased their level of physical activity and that they felt better and healthier [27]. During the exercise programme, the participants developed a more positive attitude towards exercising, and they reported that the social atmosphere and social contacts were important aspects of the intervention [27]. A change was also observed in the number of consultations with their GP, which decreased by 20% during the Big!Move project [28,29]. The projects show that a lifestyle intervention can be effective, when specific barriers are overcome. The BeweegKuur programme tries to overcome potential motivational barriers for the patients by training health care providers to apply motivational interviewing. Health care providers are supported in the implementation of the BeweegKuur intervention by the NISB, which trains all health care professionals involved to work with the intervention. The Regional Support Structure for Primary Health Care (ROS) advisor can assist them in optimizing their implementation of the BeweegKuur programme. The referral by the GP is assumed to provide an important motivation for patients to start exercising, and the adoption of a multidisciplinary approach is expected to increase the success in terms of promoting a healthy lifestyle. Inclusion of the BeweegKuur programme in the Dutch basic health insurance scheme would reduce the financial barrier to patients. Both the evidence base described above and the practice-based information gathered in step 1 of the planning process were incorporated in the draft of the prototype. For more detailed information regarding the content of the intervention, we refer to Step 3 of the planning process.

### Step 2: Determinants of (sustained) exercise adherence and improved dietary behaviour

Although many diabetics and prediabetics are able to improve their physical fitness [10], most of them fail to become more physically active and to improve their diet. Developing interventions to achieve this requires thorough insights into the main determinants of (sustained) exercise adherence and improved dietary behaviour.

These determinants of (sustained) exercise adherence and improved dietary behaviour can be classified into several domains, as demographic and psychological influences coexist with social, environmental and wider policy/legislative determinants [32]. All of these factors can, directly or indirectly, influence people's motivation to be physically active and to consume a healthy diet.

Psychological determinants of exercise and dietary behaviour have been extensively studied, often based on socio-cognitive theories such as the Theory of Planned Behaviour [33]. Concepts such as enjoyment of exercise and self-efficacy have been repeatedly found to be positively associated with physical activity [34]. Factors related to healthy food consumption include health consciousness and knowledge of the prescribed number of servings, as well as knowledge of diet-disease relationships [35,36]. Specific barriers to exercise in patients with diabetes include perceived difficulty of engaging in exercise and feelings of tiredness [37]. The central construct in this theoretical framework is the intention or level of motivation to change physical activity and/or diet.

Rather than on the *level *of motivation, Self-Determination Theory [38] focuses on the *type *of motivation. The theory assumes that motivated behaviour is based on trying to fulfil the three basic psychological needs of competence, autonomy and relatedness to others, and socio-environmental influences that support these three basic needs are expected to promote intrinsic motivation [38]. According to Deci and Ryan [38], intrinsic motivation is linked to greater productivity, cognitive flexibility, and perseverance. Generally speaking, people should have a sense of choice and feel confident about being able to meet their health-related goals. They also need to feel that they are fully responsible for initiating and maintaining healthy behaviour. Previous research showed that when patients perceive their doctor as autonomy-supportive, they report greater intrinsic self-motivation for treatment adherence [39]. In order to achieve sustained behavioural change, lifestyle interventions thus need to ensure engagement in physical activities that are intrinsically enjoyed, that participants feel competent at, and that contribute to their sense of autonomy [40]. Furthermore, lifestyle interventions need to enhance autonomous motivation for diet improvement, to facilitate better maintenance of healthy behaviour change [41,42].

People's motivations, abilities and opportunities to change their health-related behaviour may also be strongly dependent on the environments they live in [41]. This implies that optimal intervention structures are the result of an investment in enhanced facilitative environments (e.g., a solid local infrastructure in which people can be referred to various local exercise facilities), which may be regarded as a crucial feature to enable sustained behavioural change.

Theory- and evidence-based knowledge about the main determinants of sustained changes in dietary behaviour and physical activity has led to the following major change objectives [2] of our intervention. The BeweegKuur programme should:

- promote participants' autonomous motivation for behaviour change,

- inform participants about lifestyle-disease relationships and ways in which they can improve their own health status,

- increase participants' willingness to change their health-related behaviours and support their own initiatives,

- discuss difficulties in behaviour change and assist participants improve their problem solving skills, and

- promote a facilitative environment to engage in sustained physical activity and healthy dietary behaviour.

### Step 3: Intervention development

An elaborate scrutiny of the first two steps resulted in the development of a draft prototype of the BeweegKuur intervention. During the first year of the study (2008), the prototype was continuously being changed, guided by formative research to achieve further improvement [14]. At the same time, a professional development and support manual was developed, based on the experiences of experts and "experts by experience". This took place under the supervision of the Development and Professional Advancement work group, consisting of representatives from the NISB, and all relevant Dutch professional organizations in the field of health care, including NHG (Dutch College of General Practitioners), KNGF (Royal Dutch Society for Physical Therapy), LVG (Association of Organized Primary Healthcare), VSG (Dutch Association for Sports Medicine), NVDA (Dutch Association of Doctors' Assistants), TNO (Netherlands Organisation for Applied Scientific Research), NPi (Dutch Institute of Allied Health Care), LHV (National Association of General Practitioners), DNO (Diabetes and Nutrition Organisation), NDF (Netherlands Diabetes Federation) and DVN (Dutch Diabetes Association).

#### Outline of the BeweegKuur programme

The BeweegKuur intervention [1] starts and ends at the GP's practice. The practice staff are responsible for including the patient, coaching and supervising them and referring them to allied health professionals and/or local exercise coaches or a sports physician. The aim of the 12-month intervention is to facilitate transfer to existing local exercise facilities [1].

The BeweegKuur programme is open to prediabetic patients and patients with type 2 diabetes (see Table 1 for the inclusion criteria per subgroup). In addition to these criteria, eligible patients must have an inactive lifestyle (i.e. not adhering to the Dutch guideline recommendation of exercising at least half an hour for five or more days a week) and motivation for behavioural change. Exclusion criteria for the BeweegKuur are type 2 diabetes with three or more complications, type 2 diabetes with serious polypharmacy and type 2 diabetes with type 3 hypertension.

**Table 1:** The inclusion criteria per subgroup.

Subgroup	Patient profile
Subgroup A	Impaired fasting glucose (fasting glucose value (finger prick) ≥5.6 mmol/l - ≤6.0, or ≥6.1; mmol/l < 6.9)
Subgroup B	Type II Diabetes (according to 2006 definition; HbA1c ≥7.0) and/or RR > 140/90 mmHg and/or side-effects from medication
Subgroup C	Type II Diabetes well-controlled

The goal of the BeweegKuur programme is to achieve health benefits through increased physical activity and improved dietary behaviour for patients with type 2 diabetes or prediabetes. The primary objective is to promote a healthy lifestyle in terms of physical activity and dietary behaviour. Secondary objectives include the improvement of physical parameters, such as HbA1c, hypertension and BMI and the cardiovascular risk profile. Long-term objectives are the prevention of type 2 diabetes in prediabetic patients and lowering the incidence of complications in type 2 diabetes patients.

#### Theoretical and practical context of the BeweegKuur intervention

The starting point for the BeweegKuur intervention is the assumption that a permanent facilitative environment, autonomy support and involvement of counsellors play a crucial role in encouraging patients to take up and maintain positive lifestyle changes. The integration of the Motivational Interviewing counselling technique [43] with an environmental approach that facilitates opportunities to engage in lifestyle changes could thus be regarded as providing optimal conditions for long-term maintenance. The primary health care setting can be regarded as a good place to start a lifestyle intervention. In the Netherlands, GPs act as the gatekeepers to health care; they see their patients regularly and know their social environment [15]. Most diabetic patients visit their GP or the practice nurse for a check-up every three months, enabling quick selection of eligible patients for BeweegKuur and detection of relapse after inclusion in the programme. Prediabetic patients do not get these regularly check-ups.

In the BeweegKuur intervention, it is the GP who determines whether an individual is eligible for the programme. Coaching and supervision are provided by a lifestyle advisor (LSA, often the practice nurse), based on principles of Motivational Interviewing [43]. Peer feedback meetings are regularly organized for the health care providers. All medical specialists involved in the BeweegKuur programme are offered training in motivational interviewing, consisting of four eight-hour sessions, with the final session after one year. This training course is essential to improve the expertise of the professionals involved in the programme. The Motivational Interviewing course incorporates concepts from Self-Determination Theory [38].

Based on specific inclusion criteria and in close collaboration with the patient, the LSA designs an individual exercise programme, which can be attended in the existing local exercise facilities or (temporarily) under the supervision of a specialized exercise coach or physiotherapist. In addition, all participants are referred to a dietician. During the BeweegKuur programme, the GP practice remains the central location, where patients have frequent contact with the LSA about their progress in the programme and about perceived barriers.

Designed as a multi-disciplinary intervention, the BeweegKuur intervention intends to create cohesion between intervention elements and aims to achieve cooperation between partners at both national and local levels. Thus, the BeweegKuur programme contributes to the development of a solid local infrastructure for prevention. The Regional Support Structure for Primary Health Care (ROS) plays a central role in the local coordination. Good contacts within the multidisciplinary team are important. Patients should feel that they are supported by a team which is involved and committed, and that all health care providers involved aim for the same goals. It is crucial for patients' confidence in therapy that health care providers have compatible goals, provide unequivocal recommendations, and fully support, respect and know one another. The LSA is the pivot of the BeweegKuur intervention, and is responsible for unequivocal and smooth communication within the multidisciplinary team and the coordination of individual activities within the BeweegKuur programme.

The BeweegKuur programme combines individual counselling with group counselling. Group counselling can promote group cohesion, which can help motivate patients. Cohesion can be seen as a field of forces that act on the participants to stay in the group [44]. These forces depend on participants being attracted to the group and the ability of the group to mediate important goals for the participants [44]. Several studies have shown that cohesion can be a significant predictor of adherence to exercise [45-48]. For example, a study by Kwak and colleagues [45] showed that, regardless of participants' cognition about exercise, a close and bonded walking group led to higher adherence. Therefore, the BeweegKuur intervention uses group education as much as possible, for dietary behaviour as well as exercise [18].

#### Duration of the BeweegKuur programme

The BeweegKuur programme takes one year. Coaching by the LSA is gradually reduced during this year, being both more frequent and more intense at the start of the programme. The final appointment with the LSA takes place one year after the start of the intervention. It is assumed that patients who are more active and have improved their dietary behaviour over the year have increased their level of intrinsic motivation towards lifestyle changes, and will be able to maintain these changes in the long run as a result of the continuous provision of a facilitative environment (e.g., [40]).

#### The exercise settings in the BeweegKuur programme

**Figure 3:**
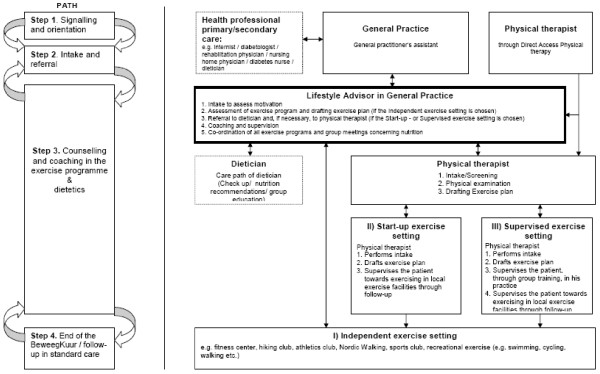
**The pathway of the BeweegKuur intervention**.

The LSA determines the intensity level of the exercise programme that best fits the individual patient. The patient can be referred to three distinct settings for physical activity (see figure 3 for the pathway of the BeweegKuur). The first is that of exercising in existing local exercise facilities (the independent exercise setting). This could include walking or cycling, in locally organised groups, with or without supervision. Further options within this setting include dancing, going to the gym or swimming. It is important for the continuation of the patients' physical activity that they intrinsically enjoy the physical activity. They also have to feel competent about engaging in the activity and it should contribute to their sense of autonomy [40]. A tailored exercise programme is designed, based on the patient's caloric expenditure. After the intake procedure and explanation of the exercise plan, the LSA and the patient meet six times during the first year (after 2, 4, 8, 16, 20 and 52 weeks) to discuss progress in terms of exercise as well as dietary behaviour and the barriers they perceived.

The second setting is a so-called 'start-up exercise setting', which is supervised by a physiotherapist for four weeks. Finally, the third setting is the 'supervised exercise setting', a three-month training programme supervised by a physiotherapist, who designs an exercise program. After the weeks of training with the physiotherapist, the LSA and the patient look for an activity that suits the patient, and the patient is referred to local exercise facilities. During the sessions with the physiotherapist, the patient starts exercising in small groups two or three times a week. The training consists of power, endurance and interval training exercises, designed by a team of specialists [49]. The group sessions can also be used for education on dietary and other behaviour, depending on the patients' preferences. The patients in the second and third settings also have six follow-up meetings with the LSA to discuss the perceived barriers and the progress in terms of exercise and dietary behaviour. The meetings with the LSA and the physiotherapist offer an autonomy-supportive environment to achieve better treatment adherence [38]. All patients in the BeweegKuur receive a logbook that assists in the self-monitoring process of the behavioural changes, and that guides and promotes the formation of Action Plans [50].

Patients are referred to one of the three settings depending on the presence of comorbidity, exercise-related complaints and strength limitations (as tested with the Cardiac Stress Test). If one or more of these criteria are met, the patient starts in the third setting, with the physiotherapist. If none of these criteria apply, the patient starts in the first setting. If the criteria are not applicable, but the patient needs to overcome certain barriers to get started, they start in the 'start-up exercise setting'.

### Step 4: Adoption and implementation plan

In the first year of the BeweegKuur project (2008), seven ROS regions started the intervention. These seven ROS regions implemented the BeweegKuur programme in 19 primary health care centres under relatively strict supervision by NISB. These primary health care centres had a positive attitude towards promoting a healthy lifestyle, and most of them were already experimenting with the implementation of preventive lifestyle interventions. In the second year, 2009, the number of participating ROS regions doubled to 14, with 41 locations. The selection of the locations was based on the experience of the advisors with the primary health care centres and their belief that a particular practice would be able to implement the intervention at relatively short notice. In 2009, the remaining six ROS regions were preparing to start, so that in 2010 all 20 ROS regions should be participating in the BeweegKuur programme and local exercise networks will be further developed. In 2012, it is expected that all inhabitants of the Netherlands will be able to participate in the BeweegKuur programme in their region if they meet the inclusion criteria. The NISB is developing a plan for further dissemination of the programme.

### Step 5: Evaluation and monitoring

The pilot project for the implementation and dissemination of the BeweegKuur intervention in the first seven ROS regions is being monitored in a formative implementation study [14]. The purpose of this study is to test the BeweegKuur intervention in terms of perceived feasibility, satisfaction and perceived efficacy of the health care providers and the patients who started the BeweegKuur in 2008. The BeweegKuur implementation process is being assessed using interviews with patients, GPs, LSAs and physiotherapists. In addition, patients receive three questionnaires, one at the start of the intervention, one after three months and one after a year. Two questionnaires are sent to the health care professionals (one at the start and one after they have worked with the intervention for 6 months). The evaluation study is based on the systematic approach proposed by Grol and Wensing [51-53] and the implementation theories proposed by Rogers [54] and Paulussen et al. [55,56]. In addition, after the pilot study, formative monitoring studies and (cost-)effectiveness studies are envisaged to examine the diffusion process and the cost-effectiveness of the intervention.

## Discussion

This paper has presented the dynamic development and implementation process of the BeweegKuur intervention. In the first two steps of the systematic intervention planning and development model used in this study, we collected all relevant information for the development of the prototype intervention. In-depth interviews, expert meetings, focus groups and reviewing previous completed and ongoing interventions yielded information about the strengths and barriers of this type of lifestyle intervention in primary care. The information showed that a lifestyle intervention can be effective, when specific barriers have been overcome. The BeweegKuur intervention tries to overcome these potential motivational barriers for both patients and health care providers. In this respect, the characteristics of the intervention, i.e. its systematic design and implementation, its theoretical underpinnings and its focus on environmental facilitation, make that the BeweegKuur has the potential to reach moderate effect sizes regarding both short-term change as well as longer-term behavioural maintenance.

There are still some aspects of BeweegKuur that need attention, however. An important point in the BeweegKuur intervention is the access to existing local exercise facilities. During the first pilot year (2008), the network of these exercise facilities was incomplete and the health care providers lacked an adequate overview of the available facilities. This is likely to have resulted in relapse by the participants in the pilot year of the BeweegKuur programme. An additional element of the relapse prevention strategy might be if GPs and LSAs address the patients' lifestyle in follow-up appointments after the end of the BeweegKuur intervention. For example, GPs and practice nurses could be advised to monitor the patients during the regular diabetes appointments. This monitoring of the patient is important, in order to quickly identify lapse and relapse.

Implementation planning for an innovation such as BeweegKuur should take the adopter categories of health care providers into account. Innovators are persons who adopt an innovation immediately after its release [54], and they are followed by the early adopters. These early adopters are characterised by a high degree of opinion leadership in most systems, and are respected and important for the adoption of the innovation by the other adopter categories [54]. The early majority is a category with a lot of informal and social contacts, and will adopt a new idea before the average system member. The late majority are sceptical about an innovation, and the final category consists of the laggards, who are characterized by a considerable resistance to the innovation [54]. In addition, a potential gap exists between the early adopters and the early majority [57]. Early adopters have other goals than the early majority, and the gap between these stages should be taken into account when implementing an innovation [57]. In the first year of the BeweegKuur project, most participating health care professionals were likely to be innovators or early adopters, as a result of the selection procedures. In the coming years of the project, the characteristics in terms of adopter categories are expected to change. The dissemination process of the BeweegKuur intervention will come to a point where the characteristics of the late majority and laggards will prevail. If the BeweegKuur programme were to be included in the basic health insurance package in the Netherlands, this may induce the late majority and laggards to join the intervention. The question remains how these adopter categories can best be approached to ensure intervention quality.

It is conceivable that the BeweegKuur intervention will be adapted to other risk groups than diabetics and prediabetics, such as COPD patients and the obese population. In fact, the prototype of the BeweegKuur programme for diabetic patients will be adapted for overweight and obese people, and will be tested in a limited number of pilot locations in 2010. After the prototype has been adjusted, the aim is to implement the intervention in more primary health care centres.

## Competing interests

The authors declare that they have no competing interests.

## Authors' contributions

SK and NV conceptualized the implementation study. JH and JM conducted the implementation study. FV and IW are responsible for the development and implementation of the BeweegKuur intervention. JH wrote the first draft of the paper. All authors have read and contributed to previous versions of the manuscript and have approved the final manuscript.
